# Population-based assessment of health, healthcare utilisation, and specific needs of Syrian migrants in Germany: what is the best sampling method?

**DOI:** 10.1186/s12874-018-0652-1

**Published:** 2019-01-07

**Authors:** Tobias Weinmann, Amal AlZahmi, Andreas Schneck, Julian Felipe Mancera Charry, Günter Fröschl, Katja Radon

**Affiliations:** 1 Occupational and Environmental Epidemiology & NetTeaching Unit, Institute and Clinic for Occupational, Social and Environmental Medicine, University Hospital, LMU Munich, Ziemssenstr. 1, 80336 Munich, Germany; 2 Department of Sociology, LMU Munich, Munich, Germany; 3 Division of Infectious Diseases and Tropical Medicine, University Hospital, LMU Munich, Munich, Germany; 4 Center for International Health, LMU Munich, Munich, Germany

**Keywords:** Epidemiologic methods, Sampling studies, Respondent-driven sampling, Sampling strategy, Recruitment strategy, Human migration, Emigration and immigration, Migrants

## Abstract

**Background:**

Studies elucidating health-related information and special needs of Syrian migrants living in Germany are urgently required. However, data is scarce and finding appropriate sampling strategies to obtain representative results is challenging. In order to increase survey response in hard-to-reach populations, new methods were developed. One of them is respondent-driven sampling (RDS), a network sampling technique. We aimed to assess if respondent-driven sampling is a better approach to recruit Syrian migrants for health research than classical random sampling via the population registry.

**Methods:**

A cross-sectional study was conducted in Munich between April and June 2017 inviting adults (18+ years) born in Syria to answer an online questionnaire asking for sociodemographic and health-related information. Recruitment of participants was done using a) random sampling via the population registry (PR) and b) RDS. The two study populations recruited via respondent-driven sampling and the population registry were compared to a sample drawn from the population registry with respect to gender and citizenship. In addition, the two study populations were compared to each other regarding self-reported health status, healthcare utilisation, lifestyle factors, social network size, and acculturation.

**Results:**

Of 374 persons randomly drawn from the population registry, 49 individuals answered the questionnaire completely (response: 13.1%) while via RDS 195 participants were recruited by 16 seeds. More persons possessed German citizenship in the total sample (20.5, 95% CI: 16.6 to 24.8%) and in the PR study population (28.6, 95% CI: 16.6 to 43.3%) than in the study population (0.5, 95% CI: 0.1 to 1.5%). Participants recruited via the population registry were older, smoked less, reported more often to hold a university degree, and indicated a higher prevalence of chronic diseases, more frequent healthcare utilisation, higher scores of acculturation as well as a larger social network compared to the study population obtained via RDS.

**Conclusions:**

Response was very low in the PR sample. The number of participants recruited via RDS was larger and led to a study population with substantially different characteristics. Our study thus indicates that RDS is a useful way to gain access to specific subgroups that are hard to reach via traditional random sampling.

**Electronic supplementary material:**

The online version of this article (10.1186/s12874-018-0652-1) contains supplementary material, which is available to authorized users.

## Background

Internal and external displacement due to the violent conflicts in the Middle East belongs to the most dramatic ongoing humanitarian emergencies in the last decades [1, 2]. Among the worst cases is the war in Syria, which not only lowered the population’s life expectancy by about 20 years but also forced a large number of people to migrate and seek refuge in other countries [3, 4]. Also after arrival in neighbouring or European host countries, migrants constitute an especially vulnerable group that suffers from a significant burden of disease [5]. They also may have special needs and specific characteristics with respect to legal status, health status, health-related risk factors, healthcare utilisation, barriers to access healthcare, or disadvantages because of low income [6–8]. Therefore, host countries urgently need to create strategies and healthcare policies to cope with this novel situation and to tailor specific interventions that meet the needs of this vulnerable population ensuring quality healthcare [1, 9, 10]. As a basis for such strategies and policies, reliable scientific evidence is vitally required [11]. In Germany, however, although being the European country hosting the largest number of Syrian migrants, the special needs of migrants have been investigated very little with studies carried out so far assessing health mainly in small samples of asylum seekers living in accommodation centres limiting the generalisability of these results [12, 13].

For the purpose of obtaining representative results in population-based cross-sectional surveys the method of choice in Germany has traditionally been random sampling via the population registries as these registries capture the population almost gapless. Also migrants are recorded in these registries shortly after their arrival. Nevertheless, it is questionable if random sampling via the population registry is the optimal method for recruiting migrants. Previous studies using traditional sampling methods like random sampling via the population registry frequently reported the problem that survey response in migrants was even lower than the currently also decreasing willingness to respond among the autochthonous population [14, 15]. Migrants are hence considered as one of the populations most hard to reach for research [16]. The low response in this sub-population could be due to language barriers, fear of the individual answers being reported to immigration authorities, lack of time or accessibility, communication difficulties, or lack of translated study instruments [17–20]. In addition, migrants frequently live hidden and under precarious conditions, partly without legal status [1]. For this reason they may not be fully covered in population registries [21], but may be captured by alternative sampling strategies.

In order to increase survey response in so-called hard-to-reach populations, new methods were developed [16]. One of them is the respondent-driven sampling strategy, a network sampling technique that starts with a small convenience sample of the target population (“seeds”) which is asked to complete the survey (potentially online) [22–24]. They are then asked to invite a limited number of their contacts (“peer-recruited participants”) who are also members of the target population by using recruitment coupons [25]. This way, the sample is expanded in recruitment waves and the dependence on the initial convenience sample is reduced [26]. Participants receive incentives for completing the survey and for successfully recruiting other respondents [25]. This double-incentive strategy increases the motivation for the seed and the peer-recruited participant to complete the survey as well as recruit further participants.

In medical research, respondent-driven sampling has been mostly used to recruit drug users [25, 27], sex workers [28, 29], and men who have sex with men [30, 31]. Beyond that, respondent-driven sampling has been shown to be an effective tool for recruiting migrants in very diverse settings and populations such as migrants from the former Soviet Union living in Poland [32], mobile migrant workers in Thailand [33], or sub-Saharan migrants in Morocco [34]. In Germany, however, so far only one study in health research employed respondent-driven sampling as sampling strategy while it has not yet been employed for sampling migrant populations [35, 36].

Therefore, as preparation for a large-scale cross-sectional study on health-related aspects of Syrian migrants living in Germany, we aimed to identify the sampling strategy most suitable for obtaining representative results. More specifically, our objective was to assess whether respondent-driven sampling is a better sampling approach to gain access to the Syrian population in Germany than traditional random sampling via the population registry. For answering this study question, we aimed at evaluating the following criteria:

- What is the total number of participants that were recruited via random sampling compared to respondent-driven sampling?
- What is the percentage of persons invited via random sampling taking part in the study (response)?
- Do the study populations recruited via random sampling and respondent-driven sampling differ from the sampling frame, the population registry, with respect to basic sociodemographic characteristics?
- Are there differences between the two study populations regarding self-reported health status, healthcare utilisation, lifestyle factors, social network size, and acculturation?

## Methods

### Study design

A cross-sectional study including migrants of Syrian origin was conducted in the period between April and June 2017 in the city of Munich, Germany. Eligible were adults (18+ years) born in Syria, residing outside refugee camps and living in Munich. The participants were recruited via two different methods: respondent-driven sampling and random sampling.

### Random sampling

According to official statistics by the local authorities, in 2017 Munich had a population of 4160 individuals with Syrian citizenship [37]. A random sample of about 10% of this population, i.e., 400 persons born in Syria and living in Munich was drawn from the population registry of the city of Munich. The registry provided each individual’s name, address, gender, and citizenship. A postal invitation letter including information about the study, data confidentiality as well as a link to the online study questionnaire was sent out to each potential participant. A first and a second postal reminder were sent six days and twenty days after the first letter, respectively.

### Respondent-driven sampling

**Fig. 1:**
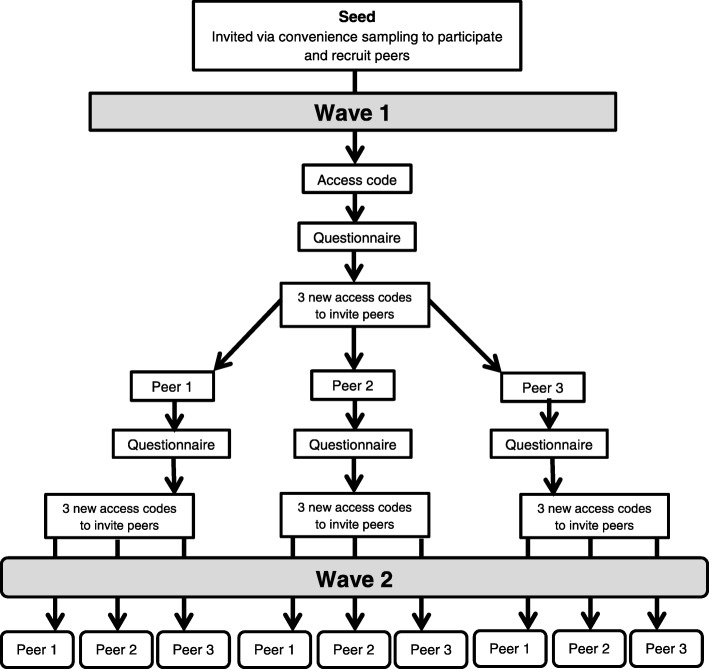
Process of recruitment of seeds and peer-recruited participants (peers) via respondent-driven sampling (RDS)

Seventeen seeds were recruited via convenience sampling at different locations: Syrian restaurants, Syrian markets, organisations working with migrants and refugees, Facebook groups, mosques, the Syrian-German Association, and university training programmes for non-German physicians. In this process, persons that reported or were reported by others to have a large number of contacts or high reputation in the community were selected as seeds. The objectives of the study, the inclusion criteria and their role as seeds were explained carefully to each seed independently. Additionally, a flyer with the link to the online questionnaire and a unique alphanumeric-access code was given to each of them. After completion of the online questionnaire, the seeds were redirected to another page where they received three new access codes to the questionnaire and were asked to recruit three of their contacts fulfilling the inclusion criteria into the study (“peer-recruited participants”). Each seed received a shopping voucher worth five euros for his or her own participation and an additional voucher for each peer-recruited participant who completed the online questionnaire. A respondent who successfully recruited three persons thus obtained incentives worth up to 20 euros (Fig. 1).

Respondent-driven sampling ended when 195 participants, including 16 of the 17 seeds, from two waves answered the questionnaire and the participation activity was sedentary for two days. One out of the 17 seeds invited failed to participate in the study. The seeds and all participants were allowed to recruit up to three new participants resulting in a recruitment chain that originates from the seed. The distance from the seed in the recruitment chain denominates the recruitment wave. The participants recruited by the seed therefore belong to the first recruitment wave. Because the seeds were collected not at random, it takes some waves to assure independence from the selective sample of seeds called equilibrium. In our data, this equilibrium was reached after one recruitment wave using the approach suggested by Heckathorn [22]. After the exclusion of all seeds and participants included in the first wave as well as single participants left from recruitment chains, 156 participants remained in the analysis sample.

### Online questionnaire

The questionnaire was based on validated instruments such as the “German Health Update” study (GEDA) and the revised sociocultural adaptation scale (SCAS-R) [39–43]. It covered the following aspects:

- Sociodemographic information (GEDA)
- Chronic diseases (GEDA)
- Healthcare utilisation (GEDA)
- Lifestyle factors (GEDA)
- Social network size
- Sociocultural adaptation (SCAS-R)

The sociodemographic section included questions on age, gender, place of birth, citizenship, employment, partnership status, marital status, and level of education. The part on chronic diseases assessed if the respondents suffered from physical complaints, i.e. feeling unwell in the last four weeks prior to the survey as a result of their physical health. They were also asked to indicate if they had been diagnosed with one of the following conditions: diabetes mellitus, arthrosis, back pain, hypertension, high cholesterol, heart attack, cancer, neurologic disorders, and asthma. Healthcare utilisation was captured by asking the respondents about the use of a general practitioner (GP) and other physicians as well as dental check-ups in the 12 months prior to the survey. With respect to lifestyle factors, respondents were asked if they did any sports or exercise in the last three months prior to the survey. Additionally, they were asked for alcohol and tobacco consumption. The social network size was defined as the total number of Syrians in Munich whom the participants know. The last part of the questionnaire covered the acculturation measures. Participants were asked to scale the interest people show in what they do and the ease of receiving help from neighbours when needed.

The questionnaire as well as all study information and the informed consent form were provided online in Arabic and English. Therefore, all the documents were translated into Arabic and English with back-translation and consistency check. The questionnaire was programmed using LimeSurvey (LimeSurvey GmbH, Hamburg, Germany). The survey was conducted anonymously in order to increase invitees’ trust in the investigation. No personal identifying information such as name or address was collected from the participants and there was no possibility to link the questionnaire data to personal data. Written informed consent was obtained from each study participant and the study was approved by the ethics committee at the Medical Faculty of LMU Munich.

### Statistical analysis

As we had received information on gender and citizenship of all 400 potential participants in the sample randomly drawn from the population registry (PR), we used these two variables as basic sociodemographic characteristics of the target population to which our two study populations were compared. Thus, in the first step we calculated absolute numbers and percentages including 95% confidence intervals (CI) for the two variables gender (female/male) and citizenship (German/Non-German) to compare the three groups population registry sample (PR sample), population registry study population (PR study population) and respondent-driven sampling study population (RDS study population).

Next, we compared the two study populations to each other with respect to their social network size (number of Syrians they know in Munich) and the sociodemographic characteristics age group (18–34 years, 35–54 years, ≥55 years), living in a steady partnership (yes/no), highest educational degree (high school degree or lower), and highest professional qualification (university degree or lower). They were also compared regarding healthcare utilisation (dental check-up in the last 12 months yes/no, use of a general practitioner in the last 12 months yes/no), lifestyle factors (current smoking yes/no, drinking alcohol yes/no, sports or exercise in the last three months yes/no), physical complaints during the last four weeks (yes/no) and life-time prevalence of physician-diagnosed chronic diseases. Regarding the latter, because of low prevalences we summarised diabetes, hypertension, high cholesterol and heart attack to “cardiovascular diseases and risks” and back pain, depression and neurologic disorders to “neurological conditions”. Asthma and cancer were excluded from the analyses because of insufficient case numbers. Concerning acculturation, the two variables other people’s interest (none/little/neither a lot nor a little/some/a lot) and ease of receiving help from neighbours (very difficult/difficult/possible/easy/very easy) were assessed.

Population registry data was analysed without applying weights while respondent-driven samplingdata was analysed by two means: 1) no weights applied and 2) weighting inversely for the number of social contacts as well as clustering by seeds to overcome bias that could result from differences in social network size [38]. Individuals with larger social network size had smaller weights compared to those with smaller social network size [38]. In addition, as classical respondent-driven samplinganalysis approaches only allow to address binary data the RDS-MOD estimator [38] was used. For categorical variables, absolute numbers (n) and percentages (%), for the continuous variable social network size unweighted and weighted means and the corresponding standard errors (SE) were calculated. Differences between the two study populations with respect to categorical variables were tested using chi-square test while Rao-Scott continuity correction for chi-square test was applied when comparing the weighted data to overcome the unequal recruitment probabilities. The difference in the social network size between the two populations was tested using a t-test. In each test, alpha was set at .05. In all calculations, only complete case analyses were performed and all statistical analyses were done with Stata 14.2.

*For the statistical analyses, only completed questionnaires were used. This was necessary because the respondent-driven sampling weights are based on the number of contacts. Without this information, no RDS estimator is computable. In order to assure the comparability of results, the restriction on completed questionnaires was also applied to the population registry study population. In the respondent-driven sampling study population only two respondents dropped out during the first two survey pages. In the PR study population, the dropout was slightly higher with five respondents. Also in this population, the majority (three subjects) dropped out during the first two survey pages.*

## Results

### Recruitment

Out of the 400 individuals randomly drawn from the population registry, 26 could not be contacted due to an invalid address. Hence, 374 potential participants were invited to the study, of which 49 answered the online survey completely (response 13.1%). Through respondent-driven sampling, using 16 seeds a total of 195 persons answered the questionnaire. As described above, 156 participants recruited by four seeds formed the study population for data analysis including all participants from the second recruitment wave onwards.

### Comparison between the population registry sample and the two study populations

With respect to gender, there were no substantial differences between the total sample drawn by the population registry (61.8% males, 95% CI: 56.8 to 66.5%), the population registry study population (65.3, 95% CI: 50.4 to 78.3%) and the respondent-driven sampling study population (56.2, 95% CI: 44.2 to 67.7%). However, statistically significantly more persons indicated to possess German citizenship in the total PR sample (20.5, 95% CI: 16.6 to 24.8%) and in the PR study population (28.6%; 95% CI: 16.6 to 43.3%) than in the RDS study population (0.5, 95% CI: 0.1 to 1.5%; Table 1).

**Table 1:** Distribution of gender and citizenship in the population registry sample and the two study populations

	PR sample (*N* = 400)			PR study population (*N* = 49)			RDS study population (*N* = 156)				
								Unweighted analysis		Weighted analysis	
	n	%	95% CI	n	%	95% CI	n	%	95% CI	%	95% CI
Gender											
Male	247	61.8	56.8 to 66.5	32	65.3	50.4 to 78.3	95	60.9	52.8–68.6	56.2	44.2 to 67.7.5
Citizenship											
German	82	20.5	16.6 to 24.8	14	28.6	16.6 to 43.3.7	3	1.9	0.4 to 5.5	0.5	0.1 to 1.5

*CI* exact Clopper-Pearson confidence interval, *PR* population registry, *RDS* respondent-driven sampling

### Comparison between the two study populations

Concerning social network size, participants recruited via the population registry reported a larger number of friends than the respondent-driven sampling study population (*p* < 0.001 in a negative binomial regression both in the weighted and unweighted RDS model). In addition, while more than 50% of the PR population were aged above 35 years, the majority of the RDS population (73.9%) was aged between 18 and 34 years (p_chi_^2^ < 0.01). Furthermore, with 53.1% (95% CI: 38.3 to 67.5%) a higher percentage of the PR study population lived in a steady partnership compared to the RDS study population (unweighted analysis: 33.3, 95% CI: 26.0 to 41.3; weighted analysis: 32.9, 95%CI: 22.4 to 44.9%) and significantly more PR participants indicated to hold a university degree (63.3, 95% CI: 48.3 to 76.6% vs. 26.1, 95% CI: 16.2 to 38.1% in the RDS sample). Regarding lifestyle factors, smoking was less frequent in the PR study population (30.6, 95% CI: 18.3 to 45.4%) than among the participants recruited through respondent-driven sampling (53.1, 95% CI: 41.3 to 64.6%; Table 2).

**Table 2:** Distribution of sociodemographic and lifestyle-related variables in the two study populations

	PR study population (N = 49)			RDS study population (N = 156)						
					Unweighted estimates			Weighted estimates		
	n	%	95% CI	n	%	95% CI	p*	%	95% CI	p**
Age							< 0.01			< 0.01
18–34	23	46.9	32.5 to 61.7	115	73.7	66.1 to 80.4		73.9	63.7 to 82.5	
35–54	13	26.5	14.9 to 41.1	39	25.0	18.4 to 32.6		25.7	17.2 to 36.0	
> 55	13	26.5	14.9 to 41.1	2	1.28	0.2 to 4.6		0.3	0.0 to 1.3	
Partnership										
Living in a steady partnership	26	53.1	38.3 to 67.5	52	33.3	26.0 to 41.3	0.01	32.9	22.4 to 44.9	0.03
Highest educational degree										
High-school degree	44	89.8	77.8 to 96.6	132	84.6	78.0 to 89.9	0.36	77.4	63.8 to 87.8	0.10
Highest professional qualification										
University degree	31	63.3	48.3 to 76.6	44	28.2	21.3 to 36.0	< 0.01	26.1	16.2 to 38.1	< 0.01
Lifestyle factors										
Sports	28	57.1	42.2 to 71.2	67	42.9	35.1 to 51.1	0.08	42.5	31.6 to 54.0	0.11
Alcohol	16	32.7	19.9 to 47.5	36	23.1	16.7 to 30.5	0.18	24.3	14.2 to 36.9	0.33
Smoking	15	30.6	18.3 to 45.4	86	55.1	47.0 to 63.1	< 0.01	53.1	41.3 to 64.6	0.01

*CI* exact Clopper-Pearson confidence interval, *PR* population registry, *RDS* respondent-driven sampling

* *p*-value calculated using chi^2^ test for the unweighted estimates of the PR and RDS study population

** *p*-value calculated using Rao-Scott correction of the Chi^2^ test for the weighted estimates of the RDS study population

Self-reports of chronic diseases showed statistically significant differences between the two groups with regards to neurological conditions with a percentage of 20.4% (95% CI: 10.2 to 34.3%) in the PR study population compared to 3.8% (95% CI: 1.0 to 9.4%) in the respondent-driven sampling population (Table 3). Concerning healthcare utilisation during the last twelve months, participants from the PR population more often indicated having undergone a dental check-up (73.5, 95% CI: 58.9 to 85.1%) and having seen a GP (73.5, 95% CI: 58.9 to 85.1%) than the respondent-driven sampling study population (dental check-ups: 31.6, 95% CI: 22.5 to 41.9%; GP visit: 19.0, 95% CI: 12.6 to 27.0%Table 3).

**Table 3:** Distribution of health-related variables in the two study populations

	PR study population (*N* = 49)			RDS study population (N = 156)						
				Unweighted estimates				Weighted estimates		
	n	%	95% CI	n	%	95% CI	p*	%	95% CI	p**
Physical complaints										
Feeling unwell in the last four weeks	16	32.7	19.9 to 47.5	18	11.5	7.0 to 17.6	< 0.01	15.8	6.6 to 29.4	0.06
Chronic diseases										
Cardiovascular	7	14.3	5.9 to 27.2	15	9.6	5.5 to 15.4	0.36	9.6	4.8 to 16.7	0.38
Neurological	10	20.4	10.2 to 34.3	5	3.2	1.0 to 7.3	< 0.01	3.8	1.0 to 9.4	< 0.01
Healthcare utilisation										
Dental check-up	36	73.5	58.9 to 85.1	58	37.2	29.6 to 45.3	< 0.01	31.6	22.5 to 41.9	< 0.01
GP visit	36	73.5	58.9 to 85.1	43	27.6	20.7 to 35.3	< 0.01	19.0	12.6 to 27.0	< 0.01

*CI* exact Clopper-Pearson confidence interval, *PR* population registry, *RDS* respondent-driven sampling

* *p*-value calculated using Chi^2^ test for the unweighted estimates of the PR and RDS study population

** *p*-value calculated using Rao-Schtt correction of the Chi^2^ test for the weighted estimates of the RDS study population

**Fig. 2:**
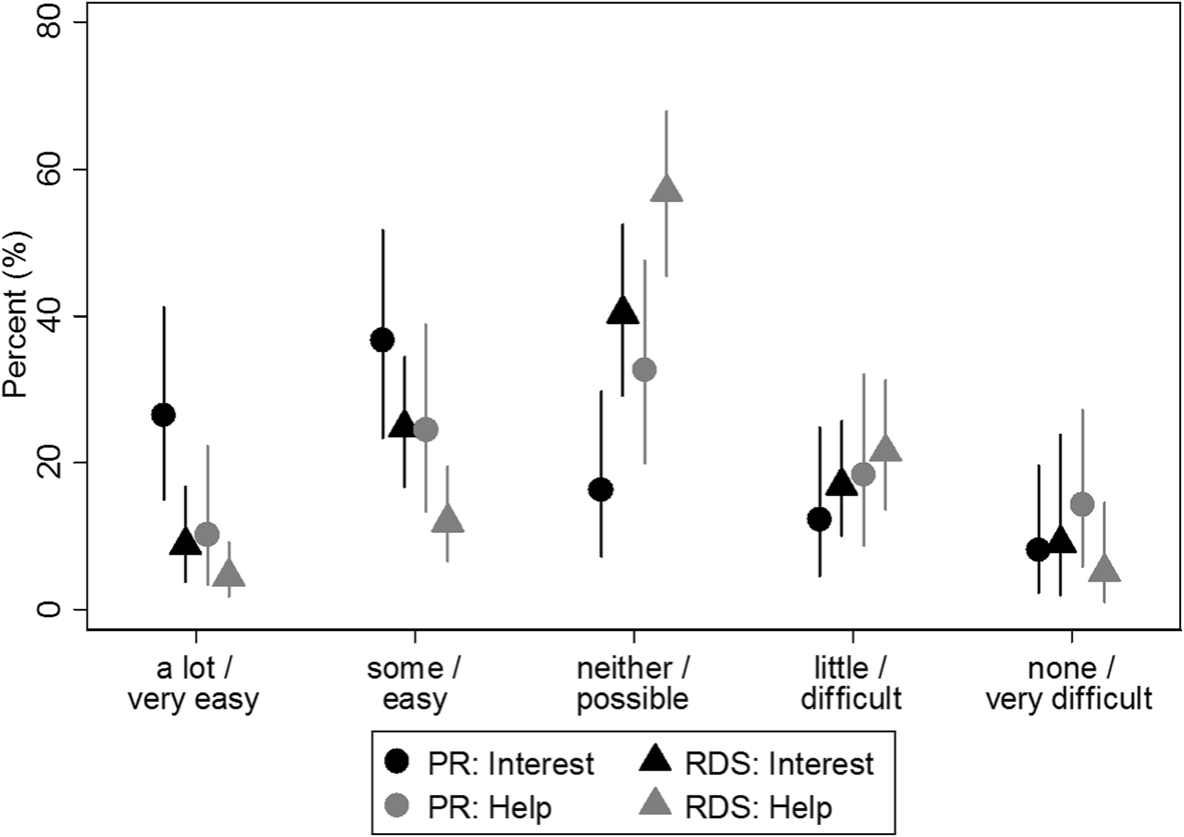
Distribution of acculturation variables in the two study populations

With regards to measures of acculturation, the PR study population and the RDS population differ in respect how much the respondents perceive they would obtain help from neighbours if needed (*p* = 0.02), but also about how they perceive others are interested in their actions (p = 0.02). In both variables the PR population is more optimistic than the RDS population indicating easier access to help or a larger interest of other people (Fig. 2).

## Discussion

The present study intended to assess the most suited sampling method for a large-scale epidemiological study investigating health-related aspects in Syrian migrants living in Germany and to evaluate if respondent-driven sampling is a better means for this undertaking than traditional random sampling. To achieve this aim, we compared random sampling and respondent-driven sampling with respect to the absolute number of recruited participants, invitees’ willingness to participate and characteristics of the study populations. Our results provide evidence that selection bias plays a major role with different sampling methods yielding study populations with different characteristics indicating that respondent-driven sampling is very helpful to get access to specific subgroups of the target population that can hardly be reached by traditional random sampling.

Via random sampling through the population registry, only a small number of participants were recruited with a very low response of 14%. This number is yet a little bit lower in comparison with the response in another German cross-sectional feasibility study including asylum-seekers [13]. Given the already mentioned general difficulty in recruiting representative samples of migrant populations, those numbers are not entirely surprising. Nevertheless, such a low response is clearly not sufficient to be able to be reasonably sure to obtain representative results as such results are most likely affected by selection bias [13]. In order to obtain more representative study samples of migrants, previous studies recommended sub-group-specific activities or participatory methods including peers in data collection [13, 15].

Using respondent-driven sampling, we applied one of the strategies that actively involve peers in the recruitment process. With the relatively small numbers of 16 seeds only, a much larger number of participants could be included via respondent-driven sampling as compared to traditional random sampling. This suggests that respondent-driven sampling is a suitable method for recruiting a reasonable number of participants with comparatively moderate effort. Having said that, the comparison between the two sampling strategies with respect to the number of recruited participants is, of course, somewhat arbitrary. Whereas in respondent-driven sampling this number depends, among other aspects, on the number of seeds, the number of participants recruited via the population registry is subject to the size of the random sample drawn. Nevertheless, we think that the aspect of absolute numbers of participants is still worthwhile to be discussed as the findings of this pilot study at least provide some information, on how many seeds we need to choose or how large the random sample needs to be to get a certain number of participants. For example, applying the above-mentioned official number of about 4000 individuals with Syrian citizenship registered in Munich to our observed response of 13% would mean that in total we could expect to recruit about 500 participants via the population registry.

As another criterion for evaluating the feasibility of our sampling methods, we compared the two study populations with total sample drawn from the population registry used as a proxy for the characteristics of the target population with respect to gender and citizenship. In the total sample obtained from the registry and in the population registry based study population a substantially higher proportion of subjects held German citizenship than in the respondent-driven sampling study population. Additionally, comparing the two study populations, participants recruited via the population registry reported more often to hold a university degree, to have a larger social network and to be better acculturated than the study population obtained via respondent-driven sampling. Moreover, population registry participants were older, smoked less and indicated a higher prevalence of chronic diseases and more frequent healthcare utilisation than respondent-driven sampling participants. That population registry participants were older could be an explanation for the health and lifestyle-related differences between the two groups. In total, these results suggest that sampling via the population registry resulted in a study population that lived longer in the host country and had a higher level of adaption to the host society while respondent-driven sampling was more suited to identify younger migrants that are less well integrated. This may be an indication of undercoverage of the population registry especially missing more recently immigrated persons.

Our findings thus imply that population-based cross-sectional surveys investigating sociodemographic and health-related aspects of migrants may yield different results depending on the sampling method. This observation is not only helpful for the interpretation of existing studies but also for the planning of future research. Following this, to not only recruit a very specific subgroup of migrants depending on the recruitment method but to be able to obtain more representative results, a combination of probabilistic and non-probabilistic sampling techniques might be a worthwhile option. Such an approach could also include additional sampling strategies as quota sampling [32], cluster sampling [44], convenience sampling methods like block-walking [45], or other non-probability sampling techniques [46]. This might also help to overcome limitations of respondent-driven sampling such as inability to access individuals who are socially isolated, non-random selection of seeds, inaccurate reporting of the network size leading to biased results, access to the online questionnaire by ineligible participants, or difficulties to recruit samples with mixed ethnic structure [36, 47, 48]. Combining probabilistic and non-probabilistic sampling methods, however, has several potential pitfalls that would need careful consideration. For instance, potential participants may be invited to the study via two or more of the selected recruitment methods. This issue could be addressed by requiring participants to create a unique personal identifier when completing the questionnaire or by screening incoming questionnaires for identical information. Furthermore, such an approach would lead to complex datasets containing data from both randomly and non-randomly selected participants. Hence, researchers would need to apply appropriate techniques such as the application of ‘pseudo-weights’ [49] and statistical software packages especially designed for such complex data [50].

As far as the authors are aware, this is the first German study exploring the feasibility of respondent-driven sampling to recruit hard-to-reach-populations for medical research. It is also one of the very first projects to implement research on health status, healthcare utilisation, and specific needs of Syrian migrants living in Germany. Scrutinising these questions is of high public health relevance as it is very likely that migration will continue to be a major issue for many societies [51]. It imposes specific challenges with respect to humanitarian aspects and healthcare [52]. The latter is exacerbated by specific difficulties with respect to communication or cultural, legal, and bureaucratic hurdles that need to be taken into account [10, 53]. Despite those difficulties, elucidating specific needs of migrants is of uppermost importance not only for developing appropriate health policy strategies but also for unveiling health inequalities and for ensuring dignified treatment of the migrants [8, 54, 55].

When interpreting our findings, it should be taken into account that we conducted our study in one of the German cities with the biggest proportion of migrants and a relatively good infrastructure of migrant organisations. Hence, we cannot be entirely sure to what extent our findings can be generalised to other locations where migrants are lower in numbers, less networked, potentially living more hidden, and thus even harder to access, especially as respondent-driven sampling suffers from difficulties in recruiting isolated and sparsely networked persons [48]. Moreover, there is the potential pitfall of individuals being inclined to participate multiple times in order to get the financial incentives. We tried to control this by the use of individual access codes and by additionally checking if incoming questionnaires contained exactly the same answers as previous questionnaires or conspicuously implausible answer patterns, e.g. always selection of the first answer option. Another limitation of respondent-driven sampling is that it does not allow calculating a response rate. Hence, we could not directly compare the response rates between the two methods, what on the other hand makes the comparison of the characteristics of the two study populations even more important. As, the main objective of our study was to evaluate sampling methods, the distribution of the variables that we measured in our sample should, however, not be taken as a valid and reliable description of the characteristics of Syrian migrants living in Germany. If at all, our numbers can be seen as very preliminary first findings. In that regard, it also needs to be mentioned that the city of Munich belongs to the wealthiest locations in Germany. Thus, the characteristics of our study population, e.g. educational level, might be different to migrants living in less prosperous regions. To get reliable information on health status and healthcare needs of Syrians and other ethnic minority groups in Germany, carefully designed representative studies are urgently needed. For the planning of such investigations our study provides important information concerning the selection of the sampling and recruitment strategy.

## Conclusions

As expected, response was very low among the random sample drawn from the population registry indicating a considerable level of selection bias. Using a relatively low number of seeds, a larger number of participants were recruited via respondent-driven sampling. The two recruitment strategies led to study populations with substantially different characteristics suggesting that population-based surveys investigating migrants’ health may provide different results depending on the sampling method. Therefore, our results indicate that respondent-driven sampling is a useful way to gain access to specific subgroups of the target population that are hard to reach via traditional random sampling. To avoid recruitment of only one very specific subgroup, future studies may consider mixed sampling approaches combining various probabilistic and non-probabilistic recruitment strategies.

## Additional file

Additional file 1: Study questionnaire. Study questionnaire in Arabic and English. (PDF 270 kb)

## Abbreviations

CI: Confidence interval
GEDA: German health update study
GP: General practitioner
OR: Odds ration
PR: Population registry
RDS: Respondent-driven sampling
SCAS-R: Revised sociocultural adaptation scale
