# Supplementary material for: Population-based assessment of health, healthcare utilisation, and specific needs of Syrian migrants in Germany: what is the best sampling method?
Source: BMC Med Res Methodol. 2019 Jan 7;19:5. doi: 10.1186/s12874-018-0652-1 (PMC6323854; doi:10.1186/s12874-018-0652-1)
Supplement: Supplementary file 1 — Study questionnaire. Study questionnaire in Arabic and English. (PDF 270 kb) [file 12874_2018_652_MOESM1_ESM.pdf]

test091531 → base 01/23/2017, 22:03

Bitte beachten: dies ist nur ein Entwurf des Fragebogens. Dieser wird derzeit auf Anraten des Datenschutzbeauftragten in LimeSurvey übertragen.

Page 01

1. How old are you? / كم هو عمرك؟

- 18-25 year old/ بين 18-25 سنة
- 26-35 year old/ بين 26-35 سنة
- 36-45 year old/ بين 36-45 سنة
- 46-55 year old/ بين 46-55 سنة
- 56-65 year old/ بين 56-65 سنة
- >65 year old/ أكثر من 65 سنة

2. Which country were you born in?/ في أي بلد ولدت؟

3. Are you...?/ هل أنت ....؟

- Male/ذكر
- Female/أنثى

4. Since when do you live in Germany?/ منذ متى تعيش في ألمانيا

5. Do you have german citizenship? هل تملك الجنسية الألمانية؟

- Yes/ نعم
- No/ لا

6. What is your citizenship?/ ماهي جنسيتك؟

7. Do you have a steady partner?/هل لديك شريك حياة ثابت؟

- Yes/نعم
- No/لا

8. What is your marital status? By married we also mean, registered life partnerships./ ما هي حالتك الإجتماعية؟ نعني بالحالة الإجتماعية العلاقة الزوجية الموثقة.

- Married, living with a spouse/ متزوج، اعيش مع الزوج
- Married, living separately/ متزوج، نعيش منفصلين
- Single/أعزب
- Divorced/ مطلق
- Widowed/أرمل

9. What is your highest academic qualification?/ ما هو أعلى مستوى أكاديمي حصلت عليه؟

- Elementary school (Hauptschule)/ ابتدائي
- Middle school (Realschule)/ متوسط أو إعدادي
- High school (Gymnasium)/ ثانوي
- Advanced/technical/university/ مستوى جامعي تقني متقدم
- No qualification/ غير حاصل على مؤهل دراسي
- Not qualification yet (still student)/ (غير حاصل على مؤهل دراسي (مازلت طالب

10. What is your highest professional qualification?/؟ما هي أعلى المؤهلات المهنية الخاصة بك

- University or higher education institution/جامعة أو مؤسسة للتعليم العالي
- University of applied sciences/جامعة العلوم التطبيقية
- Vocational school/مدرسة مهنية
- Apprenticeship/متدرب
- No qualification yet (student)/(غير مؤهل بعد (طالب
- No qualification/غيرمؤهل
- Other training certificate/شهادة تدريبية أخرى

11. Are you currently in...?/؟... هل تعمل حالياً

- Full-time employment/ عملاً منتظماً كاملاً
- Partime employment/ عملاً جزيء لبعض الوقت
- Ocassional or irregular employment/ (عملاً عرضياً (غير منتظم

12. In total how many hours a week are you employed for?/ في المجموع كم عدد ساعات عملك؟

- 0-20 hours/ بين 0-20 ساعة
- 21-40 hours/ بين 21-40 ساعة
- 41-60 hours/ بين 41-60 ساعة
- 61-80 hours/ بين 61-80 ساعة
- 81-100 hours/ بين 81-100 ساعة

13. What is the title of your main paid job? By main paid job, we mean the one where you spend most hours./ ما هو مسماك الوظيفي الأساسي الذي تحصل منه على راتب؟ المقصود بالمسمى الوظيفي الذي تحصل منه على راتب، أي الذي تمضي فيه معظم الساعات

14. What was the title of your last main paid job in Syria?/ ما هو مسماك الوظيفي لآخر وظيفة مدفوعة الراتب في سوريا؟

15. When you think about your physical health-including physical illnesses and injuries, on how many days in the last four weeks did you feel unwell as a result of your physical health?/ عند التفكير بصحتك الجسدية متضمناً ذلك الأمراض الجسدية والإصابات، كم عدد الإيام خلال الأسابيع الأربع الماضية شعرت بتوعك نتيجة صحتك الجسدية؟

- Please right the total number of days
- Never
- everyday/all the time

16. Are you severely disabled?/ هل تعاني من إعاقة شديدة؟

- Yes/ نعم
- No/ لا

17. To what extent are you permanently restricted by illness in performing your everyday activities? By permanently we mean for at least half a year. / إلى أي مدى أنت مقيد بشكل دائم بسبب المرض من أداء أنشطتك اليومية؟ المقصود بشكل دائم، أي لمدة نص سنة على الأقل.

- Significantly restricted/ مقيد بشكل كبير
- Restricted, but not significantly./ مقيد لكن ليس بشكل كبير
- Not restricted/ لست مقيد

18. Have you ever undergone medical or psychotherapy treatment for depression?/ هل خضعت سابقاً لأي علاج طبي أو علاج نفسي لمرض الإكتئاب؟

- Yes/ نعم
- No/ لا

19. Over the past 2 weeks, how often have you been bothered by any of the following problems?/ خلال الأسبوعين الماضيين، كم مرة انزعجت من أي من المشاكل التالية؟

	Not at all/ على الإطلاق	Several days/ عدة أيام	More than half of the days/ أكثر من نصف الأيام	Nearly every day/ تقريبا كل يوم
Little interesting or pleasure in doing things/قلة الإهتمام أ، الإستمتاع بأداء بعض الأمور	○	○	○	○
Feeling down, depressed or hopeless/الشعور بالإستياء أو الإكتئاب أو فقدان الأمل	○	○	○	○
Trouble falling asleep, staying asleep or sleeping too much/ صعوبة في الخلود للنوم أو البقاء نائما أو النوم لفترات طويلة	○	○	○	○
Feeling tired or having little energy / الشعور بالتعب أو نقص الطاقة	○	○	○	○
Poor apetite or overeating/ فقدان الشهية أو الإفراط في الأكل	○	○	○	○
Feeling bad about yourself or that you´re a failure or have let yourself or your family down / شعور سيء عن نفسك أو اعتبار أنك فاشل أو تحبط نفسك أوعائلتك	○	○	○	○
Trouble concentrating on things, such as reading the newspaper or watching television / صعوبة التركيز في الأشياء مثل قراءة صحيفة أو مشاهدة التلفاز	○	○	○	○
Moving or speaking so slowly that other people could have noticed, or the opposite (being so fidgety or restless that you moved around a lot more than usual) / التحرك أو التحدث ببطء شديد، لدرجة أنك لوحظت من قبل الأشخاص الآخرين، أو العكس، كونك عصبي وتقوم بأشياء كثيرة على غير المعتاد	○	○	○	○
Thoughts that you would be better off dead or of hurting yourself in some way/ التفكير بأنك قد تكون أفضل حالا لو كنت ميتاً أو آذيت نفسك بطريقة ما	○	○	○	○

20. Has a doctor ever diagnosed you with?/هل تم تشخيصك من قبل طبيب بأحد الأمراض التالية

- ☐ Asthma. Note: synonyms for bronchial asthma: allergic bronchial asthma, lung asthma, allergic asthma/ الربو
- ☐ Chronic Bronchitis/ التهاب القصبات الهوائية المزمن
- ☐ Sugar disease or Diabetes/ مرض السكري
- ☐ Kidney disease, renal insufficiency/ مرض كلوي أو فشل كلوي
- ☐ Chronic liver disease/ التهاب الكبد المزمن
- ☐ Arthritis, degenerative joint disease/التهاب المفاصل ومرض المفاصل التنكسية
- ☐ Arthrosis/تآكل العظام
- ☐ Osteoporosis/هشاشة العظام
- ☐ Chronic back pain or neck pain./آلام الظهر
- ☐ High blood pressure/ارتفاع ضغط الدم
- ☐ High cholesterol/ ارتفاع الكلسترول
- ☐ Heart Attack, coronary heart disease or Angina pectoris. / نوبة قلبية
- ☐ Myocardial insufficiency or heart failure/قصور عضلة القلب أو فشل القلب
- ☐ Stroke/ السكتة الدماغية
- ☐ Cancer or Malignant tumor/السرطان أو الورم الخبيث
- ☐ Depression/ الإكتئاب
- ☐ Chronic neurologic disorders/الاضطرابات العصبية المزمنة

21. Are you currently taking drugs for?/ هل تتناول حاليا دواءً لأي من الأمراض التالية؟

- ☐ High cholesterol/ ارتفاع الكلسترول
- ☐ High blood pressure/ ارتفاع ضغط الدم
- ☐ Diabetes/ السكري

22. Do you use glasses or contact lenses?/ هل تستخدم نظارات أو عدسات لاصقة

- ○ Yes/نعم
- ○ No/لا

23. Do you use a hearing aid?/ هل تستخدم أجهزة مساعدة للسمع؟

- ○ Yes/ نعم
- ○ No/ لا

Page 05

24. Have you suffered a traumatic event in the past?

- ☐ yes
- ☐ no

25. If yes, please indicate which of the of the following events/ نرجو أن تذكر إن كنت قد تعرضت لأي من الحوادث التالية

You can choose more than one option./ يمكنك اختيار أكثر من إجابة.

- [ ] Opressed because of ethnicity, religion or sect/هل تعرضت للاضطهاد بسبب عرقك، دينك أو مذهبك
- [ ] You were present while someone searched for people or things in your home/هل تم تفتيش دارك بحضورك بحثاً عن أشخاص أو أشياء
- [ ] your person (your clothes and/or body) were searched/هل فُتشت أنت
- [ ] Property looted, confiscated or destroyed/هل تم نهب ممتلكاتك الشخصية أو مصادرتها أو تدميرها
- [ ] Forced to leave your hometown and settle in a different part of the country with minimal services/هل فرض عليك ترك مدينتك والسكن في منطقة أخرى تقل فيها الخدمات
- [ ] Imprisoned/هل سجنت
- [ ] Suffered ill health without access to medical care or medicine/هل عانيت من عدم إمكانية الحصول على الرعاية الطبية أو الدواء خلال مرضك
- [ ] Suffered from lack of food or clean water/هل عانيت من عدم وجود الطعام والماء الصافي
- [ ] Forced to leave your country/هل اضطررت على الهرب من وطنك
- [ ] Expelled from your country based on ancestral origins, religion or sect/هل فرضت عليك الهجرة من وطنك بناءً على أصل أجدادك، دينك أو مذهبك
- [ ] Lacked shelter/ هل لم يكن لديك مأوى
- [ ] Witnessed the desecration or destruction of religious shrines or places of religious instruction/هل شاهدت انتهاك حرمة أو تدمير العتبات المقدسة أو المراكز التعليمية الخاصة بدينك أو مذهبك
- [ ] Witnessed the arrest, torture, or execution of religious leaders or important member of the tribe/هل شاهدت اعتقال أو تعذيب أو اعدام شخصيات مهمه من عشيرتك، دينك أو طائفتك
- [ ] Witnessed mass execution of civilians/هل شاهدت إعداماً جماعياً للمدنيين
- [ ] Witnessed shelling, burning, razing of residential areas or marshlands/ هل شاهدت قصف أو إحراق أو تدمير الأماكن السكنية أو الأهوار
- [ ] Witnessed chemical attacks on residential areas or marshlands/ هل شاهدت هجمات كيميائية على المناطق السكنية أو الاهوار
- [ ] Witnessed rotting corpses/ هل شاهدت جثثاً متعفنة
- [ ] Exposed to combat situations (explosions, artillery fire, shelling) or landmine/هل تعرضت لميدان الحرب (انفجارات، قصف مدفعي، رمي الأسلحة) أو الألغام
- [ ] Serious physical injury from combat situation or landmine/هل أصبت إصابة جسمية خطيرة بسبب التعرض لميدان الحرب والألغام
- [ ] Used as human shield/هل استُخدِمت كدرع بشري
- [ ] Serious physical injury of family member or friend from combat situation or landmine/هل أصيب أحد أفراد عائلتك أو أصدقائك إصابة جسمية خطيرة بسبب التعرض لجو الحرب والألغام
- [ ] Confined to home because of chaos and violence outside/هل أجبرت على البقاء في الدار بسبب الفوضى والعنف في الخارج
- [ ] Witnessed someone being physically harmed/(هل شاهدت شخصاً ما يتعرض للأذى الجسمي (الضرب، الطعن، ...إلخ
- [ ] Witnessed sexual abuse or rape/هل شاهدت عملية الإساءة الجنسية أو الاغتصاب
- [ ] Witnessed torture/هل شاهدت حالة تعذيب
- [ ] Witnessed murder/هل شاهدت حالة قتل
- [ ] Forced to inform on someone placing them at risk of injury or death/هل أجبرت على الإخبار عن شخص آخر مما عرضه لخطر الإصابة أو الموت
- [ ] Forced to destroy someone´s property/هل أجبرت على تدمير ممتلكات شخص آخر
- [ ] Forced to physically harm someone/هل أجبرت على إلحاق الأذى الجسمي (الضرب، الطعن،..إلخ) بشخص ما
- [ ] Murder or violent death of family member/ هل قتل أحد من أفراد عائلتك (طفلك، زوجك،..إلخ) أو مات نتيجة العنف
- [ ] Murder or violent death of friend/هل قتل صديقك أو مات نتيجة العنف
- [ ] Forced to pay for bullet used to kill family member/(هل أجبرت على دفع قيمة الطلقة المستخدمة لقتل أحد أفراد عائلتك (طفلك، زوجك،.. إلخ
- [ ] Received the body of a family member and prohibited from mourning them and performing burial rites/هل استلمت جثة أحد أفراد عائلتك (طفلك، زوجك،.. إلخ) ومنعت من البكاء وإقامة مراسيم الدفن عليه
- [ ] Disappearence of a family member/(هل اختفى أحد أفراد عائلتك (طفلك، زوجك،...إلخ
- [ ] Disappearence of a friend/هل اختفى صديقك

- Family member kidnapped or taken as hostage/هل اختطف أحد أفراد عائلتك (طفلك، زوجك،...إلخ) أو أخذ كرهينة
- Friend kidnapped or taken as hostage/هل اختطف صديقك أو اخذ كرهينة
- Someone informed on you placing you and your family at risk of injury or death/هل بلّغ أحد عنك مما عرضك وعائلتك لخطر الإصابة أو الموت
- Physically harmed./(هل تعرضت للأذى الجسمي (الضرب، الطعن،...إلخ)
- Kidnapped or taken as hostage./ هل اختطفت أو أخذت كرهينة
- Sexually abused or raped/هل تعرضت للإساءة الجنسية أو اغتصبت
- Tortured/هل تم تعذيبك (بمعنى أثناء وجودك في الأسر تعرضت إلى المعاناة النفسية أو الجسدية بشكل متعمد ومنتظم

26. Please specify any other situation that was very frightening or in which you felt your life was in danger./ نرجو أن تحدد أي مواقف أخرى مخيفة أو شعرت عندها بأن حياتك معرضة للخطر

27. The following are symptoms that people sometimes have after experiencing hurtful or terryfying events in their lives. Please read each one and decide how much the symptoms bothered you in the last week./ إن الأعراض التالية هي أعراض يشعر بها أحيانا الأشخاص الذين تعرضوا لحوادث مؤلمة أو مفزعة في حياتهم. الرجاء قراءة كل بند بدقة، وتحديد مدى معاناتك من هذه الأعراض خلال الأسبوع المنصرم.

	Not at all/ لا أبداً	A little/قليلاً	Quite a bit/ إلى حد كثير	Extremely/ بشدة
Recurrents thoughts or memories of the most hurtful or terrifying events/هل تعاودك الذكريات والأفكار لأكثر الحوادث ألما أو فزعا	○	○	○	○
Feeling as though the event is happening again/هل تشعر وكأنك تعيش الحادثة مرة أخرى	○	○	○	○
Recurrent nightmares/هل تأتيك كوابيس (أحلام مفزعة) متكررة	○	○	○	○
Feeling detached or withdrawn from people/هل تشعر بالإنفصال أو الإنعزال عن الناس	○	○	○	○
Unable to feel emotions/هل تجد نفسك غير قادر على الإحساس بالعواطف	○	○	○	○
Feeling humpy, easily startled/هل تجد نفسك شديد الجفلان أو الإستثارة	○	○	○	○
Difficulty concentrating/هل تجد صعوبة في تركيز أفكارك	○	○	○	○
Trouble sleeping/هل تجد صعوبة في النوم	○	○	○	○
Feeling on guard or vigilant/هل تجد نفسك متوجسا أو على حذر	○	○	○	○
Feeling irritable or having outbursts of anger/هل تجد نفسك سريع الإنفعال أو تنتابك سورات من الغضب	○	○	○	○
Avoiding activities that remind you of the hurtful event/هل تتجنب الأعمال التي تذكرك بالحادثة المؤلمة	○	○	○	○
Inability to remember parts of the most hurtful events/هل تجد نفسك غير قادر على تذكر بعض الحوادث التي سببت لك أشد الألم	○	○	○	○
Less interest in daily activities/هل تجد نفسك أقل اهتماماً بالأعمال اليومية	○	○	○	○
Feeling as if you don´t have a future/هل تشعر وكأنه لا مستقبل لك	○	○	○	○
Avoiding thoughts or feelings associated with the hurtful events/هل تتجنب الأفكار أو المشاعر المرتبطة بالحوادث المؤلمة	○	○	○	○
Sudden emotional or physical reaction when reminded of the most hurtful events/ هل تشعر برد فعل جسمي أو عاطفي عند تذكيرك بالحوادث المؤلمة	○	○	○	○

28. When you drink alcohol, how many alcoholic beverages do you usually drink in a day? By an alcoholic drink we mean a small bottle of beer (0.33l), a small glass of wine (0.125l), a glass of champagne, a double schnapps, liquor or a bottle of alcopops./ عندما تشرب الكحول، كم هي كمية المشروبات الكحولية التي تشربها في اليوم الواحد؟

- 1 to 2 alcoholic drinks/ ١ إلى ٢ مشروب كحولي
- 3 to 4 alcoholic drinks/ ٣ إلى ٤ مشروب كحولي
- 5 to 6 alcoholic drinks/ ٥إلى ٦ مشروب كحولي
- 7 to 9 alcoholic drinks/ ٧ إلى ٩ مشروب كحولي
- 10 or more alcoholic drinks/ ١٠ أو أكثر
- I drink alcohol less frequently than everyday/ أشرب الكحول بكمية أقل من كل يوم
- I don’t drink alcohol/ أنا لا أشرب الكحول

29. Do you currently smoke- even if it is only occasionally?هل تدخن حالياً، حتى لو كان فقط في بعض الأحيان؟

- Yes, daily./ نعم، بشكل يومي
- Yes, occasionally./ نعم، في بعض الاحيان
- No, not anymore./ لا، ليس بعد الآن
- I had never smoked./ لم أدخن قط

30. Have you attended a dental check-up in the last 12 months? هل حضرت فحص أسنان في الأشهر12 الماضية؟

- Yes/نعم
- No/ لا

Page 07

31. When did you last consult a doctor, not including dentists?/ متى هي آخر مرة استشرت فيها طبيب، غير طبيب الأسنان؟

- During the last 4 weeks./ في الأسابيع الأربعة الماضية
- 1 to 3 months ago./ من 1 إلى 3 شهور الماضية
- 4 to 12 months ago./ من4 إلى 12 شهر الماضي
- 1 to 5 years ago./ من 1 إلى 5 سنوات الماضية
- More than 5 years ago./ أكثر من 5 سنوات مضت

32. Do you have a family doctor who you would normally go to first in the event of health problems?/هل عندك طبيب عائلة ترجع إليه عند تعرضك لوعكة صحية؟

- Yes/ نعم
- No/ لا

33. How many nights have you spent in hospital in the last 12 months? Note: we mean as a patient, not as a companion./ كم عدد الليالي التي أمضيتها في المستشفى في الأشهر12 الماضية؟ ملاحظة، نقصد بدخولك للمستشفى كمريض وليس كمرافق.

nights / ليلة

Page 08

34. Think about the last three months. Have you done any sport or exercise?/ فكر في الشهور الثلاثة الماضية، هل مارست أي نوع من الرياضة؟

If the answer is “No” please go to question “35 “.

- Yes/ نعم
- No/لا

35. For how many hours do you exercise or do sports a week?/ كم ساعة اسبوعياً تمارس الرياضة؟

- More than 4 hours/ اكثر من أربع ساعات
- 2 to 4 hours / ساعتان إلى إربع ساعات
- 1 to 2 hours/ ساعة إلى ساعتان
- 1 hour or less/ ساعة أو أقل

Page 09

36. How many Syrians living in Munich do you know?/ كم تعرف من السوريين الذين يعيشون في ميونخ؟

37. During the last 4 weeks, to how many of them did you have contact?/ خلال الأسابيع الآربعة الماضية، كم عدد الذين تواصلت معهم من هؤلاء؟

38. How many people are so close to you that you can rely on them if you have great personal problems?/ كم عدد الأشخاص المقربين منك والذين يمكن أن تعتمد عليهم عند مرورك في مشاكل شخصية كبيرة؟

- None/ لا يوجد
- 1 to 2/ من 1 إلى 2
- 3 to 5/ من 3 إلى 5
- More than 5/ أكثر من 5

39. How much interest and concern do people show in what you do?/ كم هي كمية الإهتمام التي يوليها الناس لما تقوم به من أعمال

- A lot/ كثيراً
- Some/بعض الشيء
- Neither a lot nor a little./ لا كثيراً ولا قليلاً
- Little./ قليل
- None/ غير مهتمون

40. How easy it is for you to get practical help from neighbors if you should need it?/ ما مدى سهولة حصولك على مساعدة عملية من جيرانك إذا كنت بحاجة للمساعدة؟

- Very easy/ سهل جداً
- Easy/ سهل
- Possible/ ممكن
- Difficult/ صعب
- Very difficult./ صعب جداً

41. Living in a different culture often involves learning new skills and behaviors. Thinking about life in Germany, please rate your competence at the following behaviours from (1) not at all competent to (5) extremely competent. / المعيشة في ثقافة مختلفة عادة ما تتضمن تعلم مهارات وسلوكيات جديدة. بالتفكير عن السكن في ألمانيا، يرجى منك تقييم كفاءتك في السلوكيات التالية: من (1) غير مؤهل على الإطلاق إلى (5) مؤهل بشكل كبير

	1. Not at all competent/ غير مؤهل على الإطلاق	2	3	4	5. Extremely competent/ مؤهل بشكل كبير
Building and maintaining a relationship/ بناء والحفاظ على علاقة	○	○	○	○	○
Obtaining community services I require/ الحصول على الخدمات الإجتماعية التي أحتاجها	○	○	○	○	○
Understanding and speaking German./ فهم والتحدث باللغة الألمانية	○	○	○	○	○
Adapting to the population density./ التكيف مع الكثافة السكانية.	○	○	○	○	○
Finding my way around./ العثور على مساراتي المطلوبة لقضاء احتياجاتي	○	○	○	○	○
Changing my behavior to suit social norms, rules, attitudes, beliefs, and customs/ تغيير سلوكياتي لتتناسب مع القواعد والعادات والقيم والمعتقدات الإجتماعية	○	○	○	○	○

Page 10

Last Page

Thank you for completing this questionnaire!

We would like to thank you very much for helping us.

Your answers were transmitted, you may close the browser window or tab now.

Dr. Amal AlZahmi, Institute and Outpatient Clinic for Occupational, Social and Environmental Medicine, Faculty of Medicine
